# Toxicity Assessment of Iron Oxide Nanoparticles in Zebrafish (*Danio rerio*) Early Life Stages

**DOI:** 10.1371/journal.pone.0046286

**Published:** 2012-09-27

**Authors:** Xiaoshan Zhu, Shengyan Tian, Zhonghua Cai

**Affiliations:** 1 Division of Ocean Science and Technology, Graduate School at Shenzhen, Tsinghua University, Shenzhen, People's Republic of China; 2 College of Marine Science and Engineering, Tianjin Key Laboratory of Marine Resources and Chemistry, Tianjin University of Science and Technology, Tianjin, People's Republic of China; University of Illinois at Chicago, United States of America

## Abstract

Iron oxide nanoparticles have been explored recently for their beneficial applications in many biomedical areas, in environmental remediation, and in various industrial applications. However, potential risks have also been identified with the release of nanoparticles into the environment. To study the ecological effects of iron oxide nanoparticles on aquatic organisms, we used early life stages of the zebrafish (*Danio rerio*) to examine such effects on embryonic development in this species. The results showed that ≥10 mg/L of iron oxide nanoparticles instigated developmental toxicity in these embryos, causing mortality, hatching delay, and malformation. Moreover, an early life stage test using zebrafish embryos/larvae is also discussed and recommended in this study as an effective protocol for assessing the potential toxicity of nanoparticles. This study is one of the first on developmental toxicity in fish caused by iron oxide nanoparticles in aquatic environments. The results will contribute to the current understanding of the potential ecotoxicological effects of nanoparticles and support the sustainable development of nanotechnology.

## Introduction

Manufactured nanomaterials, defined as materials with at least one dimension between 1 nm and 100 nm [1], possess enhanced or even unique physicochemical properties, such as nanoscale size effects, quantum effects, increased surface area, and higher surface curvature as well as unique electric, thermal, mechanical, and imaging properties [2], [3]. These excellent characteristics promise them a wide variety of applications and an increasing production. As of the end of 2011, more than 1300 nanotechnology products had appeared on the market [http://www.nanotechproject.org/inventories/consumer/analysis_draft/. Accessed 02/25/2012]. And, it is estimated that nanotechnology will generate a market worth US$1 trillion by 2015 [4]–[6].

Iron oxide nanoparticles (NPs), with the main forms being of magnetite (Fe_3_O_4_) and hematite (α-Fe_2_O_3_ and γ-Fe_2_O_3_), have attracted extensive interest for application because of their superparamagnetic properties and high catalytic abilities [7], [8]. The related fields for the application of iron oxide NPs include terabit magnetic storage devices, pigments, catalysis, sensors, high-sensitivity biomolecular magnetic resonance imaging, tumor therapy, drug and gene transfer to cells, and labeling of macromolecules and cells [9]–[12]. Moreover, iron oxide NPs can be used as an adsorbent in the removal of metals from aqueous solutions [13]–[15]. A catalytic property of iron oxide NPs, which is widely used in laboratory tests and in the treatment of wastewater, has also been reported recently [16]. The increasing production and use of iron oxide NPs will inevitably result in a greater exposure risk for both people and the environment. Thus, it has become essential to assess the potential health and environmental effects of iron oxide NPs on humans, non-human biota, and ecosystems.

Until recently, most studies on the potential effect or toxicity of iron oxide NPs have focused on mammals (such as mice and rats) and/or on different types of cell lines [17]–[21]. However, to date, very few studies have investigated the ecotoxicity of iron NPs, particularly in aquatic systems [22]–[24]. Zhu et al. (2008) were the first to report that pumpkin plants (*Cucurbita maxima*) grown in an aqueous medium containing iron oxide (Fe_3_O_4_) NPs could absorb, translocate, and accumulate NPs in the plant tissues [25]. More recently, Nations et al. (2011) have reported that iron oxide (Fe_2_O_3_) NPs decreased the snout-vent length (SVL) of *Xenopus laevis* tadpoles at a concentration as low as 0.001 mg/L. The SVL increased at 1 mg/L Fe_2_O_3_ NPs and then steadily decreased at higher concentrations (10, 100, and 1000 mg/L). The total body length of *X. laevis* tadpoles exposed to 1000 mg/L Fe_2_O_3_ NPs was also significantly reduced (*p*≤0.033) compared with controls [23]. In addition, García et al. (2011) conducted a series of acute ecotoxicity tests, including phytotoxicity using several plant seeds, aquatic toxicity using *Daphnia magna*, and a bioluminescent test (Microtox®) using the bioluminescent marine bacterium *Vibrio fischeri* as a model organism. Their results showed that iron oxide (Fe_3_O_4_) NPs (≤0.67 mg/mL) exhibited low or no toxicity on plant seeds. However, *D. magna* (LC_50_ = 23×10^−4^ mg/mL) and *V. fischeri* (EC_50_ = 0.24 mg/mL) demonstrated extreme sensitivity to iron oxide NPs, which indicates the high toxicity of iron oxide NPs in aquatic environments [24]. Another form of iron oxide NPs, Fe_2_O_3_ NPs, even at a low concentration of 1 mg/L, was also able to affect the hematological, biochemical, ionoregulatory, and enzymological parameters in an Indian major carp, *Labeo rohita*, upon a 96-h static exposure [26]. This finding suggests a high potential toxicity of iron oxide NPs in aquatic environments [26]. The above pioneering studies indicate that the release of iron oxide NPs into the environment may be harmful to various eco-relevant organisms, which highlights the need for further research into the environmental impact and biological effects of iron oxide NPs.

To further assess the ecological effect, especially the aquatic toxicity, of iron oxide NPs, we conducted an early life stage (ELS) test using zebrafish (*Danio rerio*) embryos and larvae as model organisms. The ELS test is currently one of the most widely used tools in environmental science research, especially for investigating the toxicity and teratogenicity of chemicals that could significantly affect environmental and human health [27], [28]. The present study also aimed to assess whether conventional standardized tests, such as the ELS test, are useful in determining the ecotoxicity of NPs when no or insufficient data are available.

## Materials and Methods

### Iron oxide NPs and characterization

Uncoated alpha-Fe_2_O_3_ (α-Fe_2_O_3_) nanoparticles (nFe_2_O_3_) with a published particle size of 30 nm were purchased from Nanjing High Technology NANO CO., Ltd. (Nanjing, China). Supplied as a red powder, the nanoparticles had a purity of ≥99.5% and a specific surface area of 38.57 m^2^/g. Stock solutions (1000 mg/L) of nFe_2_O_3_ were prepared by stirring nFe_2_O_3_ vigorously in zebrafish culture medium (consisting of 64.75 mg/L NaHCO_3_, 5.75 mg/L KCl, 123.25 mg/L MgSO·7H_2_O, and 294 mg/L CaCl_2_·2H_2_O) prepared according to International Organization for Standardization (Geneva, Switzerland) standard 7346-3:1996 [29], using a magnetic agitator at room temperature for 2 h. The morphology of nFe_2_O_3_ was observed under a transmission electron microscopy (TEM, Hitachi H-7650, Japan).

The actual size distributions of nFe_2_O_3_ in the culture medium were determined using a dynamic light scattering device (DLS; Brookhaven Instrument Corporation, Holtsville, NY, USA). Before carrying out the DLS measurements, no sound or ultrasound was applied to agitate the particles in the culture medium. Suspensions (10 mg/L) were introduced into a polystyrene disposable cuvette; and the size measurements were conducted immediately according to the manufacturer's guidelines. All DLS measurements were performed at 26°C, which was set in accordance with the water temperature in the exposure experiments. Thereafter, the average size of the nFe_2_O_3_ aggregates was documented.

### Zebrafish culture and embryo selection

The detailed procedure for zebrafish culture and embryo selection is provided in a previous paper [28]. In brief, zebrafish adults with a roughly 2∶1 male/female sex ratio were kept in a 250-L full glass aquarium under the following conditions: 26°C±1°C, 14-h/10-h light/dark cycle. Spawning was triggered once the light was turned on in the morning and completed within 30 min. At 4–5 h post-fertilization (hpf), embryos were collected and rinsed several times with the culture medium to remove residues on the egg surface. Healthy embryos at the blastula stage were then selected for subsequent experiments.

### Exposure process

Test solutions were prepared immediately prior to use by diluting the stocks of nFe_2_O_3_ with the culture medium. During the preparation of the diluted solution, the stock solution/mixture was continuously stirred with a magnetic stirrer to maintain the suspension at as stable a concentration as possible. The embryo toxicity test design was followed according to a standard guideline [30] and the methods of Schulte and Nagel [31]. The embryo toxicity test was initiated as soon as the intact fertilized eggs were selected. Twenty-four eggs (blastula stage) were transferred to the test wells of a 24-well multiplate (Costar® 24Well Cell Culture Cluster, Corning Incorporated, NY, USA). Twenty wells were prepared with 2 mL nFe_2_O_3_ test solution (treatment) each. The remaining four wells (control) were prepared similarly, with the culture medium replacing the test solution. The concentration gradients of nFe_2_O_3_ tested in this study were 100, 50, 10, 5, 1, 0.5, 0.1 mg/L, and the water control. The experiment was performed in triplicate (i.e., 12 embryos were used in the water control and 60 embryos in the exposure group) for each treatment. The wells were covered with transparent plastic films to ensure a constant concentration. All the plates containing experimental embryos were placed in a fish room with controlled light and temperature conditions (i.e., 26°C±1°C with a 14-h/10-h light/dark cycle). At the end of the experiment, water samples were collected and immediately tested for quality assessment. All the experimental protocols were approved by the Animal Welfare and Ethics Committee of Tsinghua University (Shenzhen), China (No. 2012-XSZ-F58).

### Embryo-larval toxicity test

Throughout the whole exposure period after fertilization, the development status of the zebrafish embryos and larvae was observed under an inverse microscope (×10–40, DMLL, Leica Corp., Germany) and documented at specified time points (*t* = 6, 12, 24, 36, 48, 60, 72, 84, 96, 120, 144, and 168 h). The endpoints used to assess developmental toxicity included embryo/larva survival and embryo hatching rate. Malformations were described and documented among the embryos and larvae from both the control and treated groups. Inhibitory tendencies were also noted and described among the embryos and larvae from both the control and treated groups using a stereomicroscope (×0.67–6.7, Olympus, Japan).

### Statistical analysis

All experiments were repeated three times independently. Data were recorded as the mean with the standard deviation (SD). For the embryo/larval bioassays, a one-way analysis of variance (ANOVA) with Tukey's multiple comparisons was used to detect significant differences between the control and treated groups. A *p*<0.05 was considered statistically significant. The EC_50_ (hatching delay) and LC_50_ (mortality) values as well as their associated 95% confidence intervals (95% CI) were calculated using a tsk method (US EPA Tsk Analysis Program, Ver.1.5 http://www.epa.gov/nerleerd/stat2/tsk.zip). The no observed effect concentration (NOEC) value was designated as the highest tested concentration that had no statistically significant effect within the exposure period when compared with the control.

## Results and Discussion

### Characterization of nFe_2_O_3_ NPs

**Figure 1:**
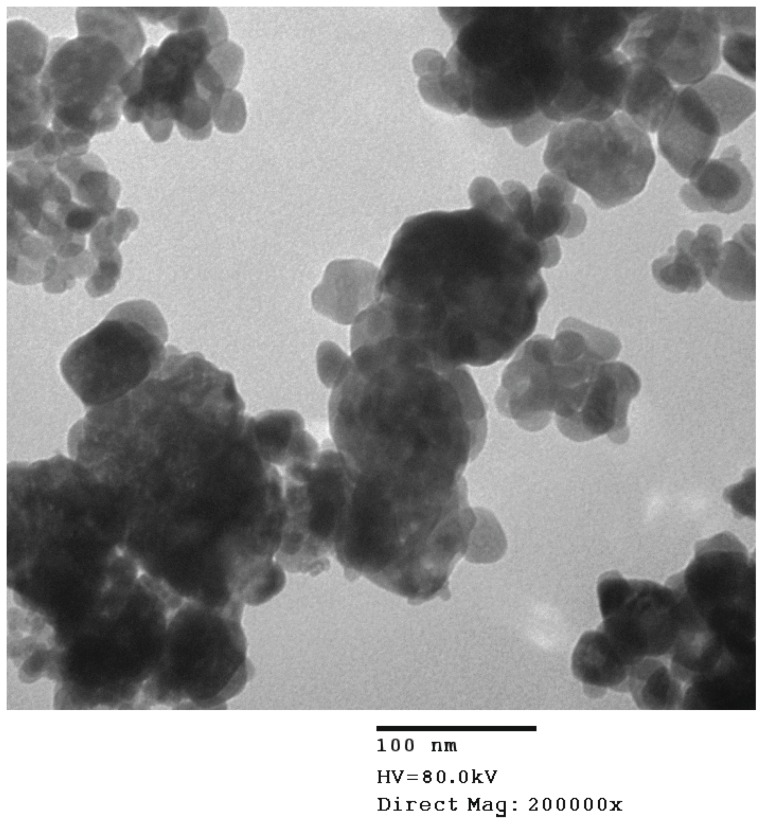
The transmission electron microscope (TEM) image of nFe_2_O_3_ aggregates (10 mg/L) in zebrafish culture medium.

In this study, the addition of nFe_2_O_3_ to the culture medium induced the formation of aggregates. Figure 1 shows a confirmatory TEM image of the large aggregations of nFe_2_O_3_ at concentration of 10 mg/l. Further determined by DLS, the average size of the nFe_2_O_3_ aggregates was calculated to be 1.025 µm, which is far larger than the nFe_2_O_3_ primary particle size of 30 nm. Moreover, these nFe_2_O_3_ aggregates were found to settle out of the water column rapidly. We did not monitor the sedimentation rate, but after 24 h a layer of nFe_2_O_3_ aggregates was observed at the bottom of the exposure well in the 24-well culture multiplate. The solution above was clearer, and it appeared as though all nFe_2_O_3_ aggregates had settled out.

### Survival

**Figure 2:**
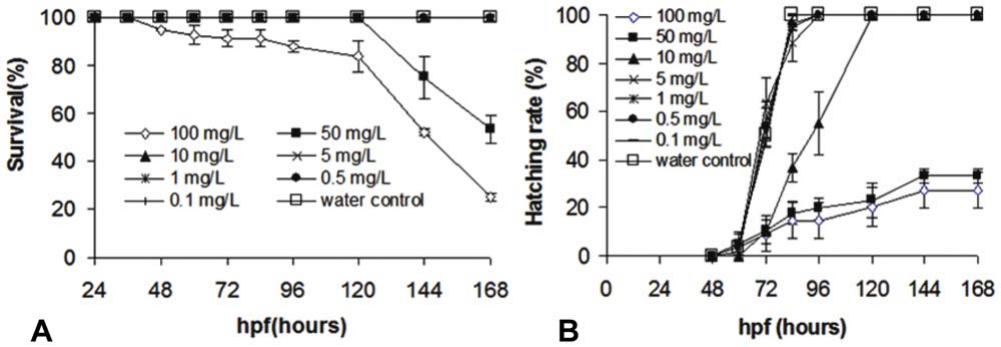
Survival (A) and Hatching rate (B) of zebrafish embryos exposed to different concentration of nFe_2_O_3_ over 168 h. Error bars represent ± one standard deviation from the mean of three replicates.

The survival of zebrafish embryos and larvae exposed to different concentrations of nFe_2_O_3_ was determined at specific time points. As shown in Figure 2A, 0.1 mg/L to 10 mg/L of nFe_2_O_3_ exhibited no toxicity to zebrafish embryos or larvae. Both 50 and 100 mg/L of nFe_2_O_3_ demonstrated toxicity, killing 75% and 45%, respectively, of the zebrafish embryos/larvae at the end of the exposure (168 hpf). As a result, the 168-h NOEC of nFe_2_O_3_ was less than 50 mg/L, and the 168 h LC_50_ was calculated to be 53.35 mg/L (95% CI: 28.21–100.89). Moreover, survival of the embryos was more than 90% at 48 hpf, but it declined sharply to 25% at 168 hpf. This result suggests that the zebrafish development toxicity of nFe_2_O_3_ is time and dose dependent.

### Hatching rate

The hatching rates of zebrafish embryos exposed to different concentrations of nFe_2_O_3_ at different development stages are shown in Figure 2B. The nFe_2_O_3_-treated embryos showed a dose-dependent effect on the hatching rates under laboratory conditions. Compared with the control group, 0.1–5 mg/L of nFe_2_O_3_ did not significantly affect the hatching rate during the 168-h exposure time. However, ≥10 mg/L of nFe_2_O_3_ displayed significant (*p*<0.05) embryo-hatching delay and toxicity. Based on this result, the 168-h NOEC and EC_50_ of nFe_2_O_3_ on the hatching rate were calculated to be 10 mg/L and 36.06 mg/L (95% CI: 20.63–63.02), respectively.

### Malformations

**Figure 3:**
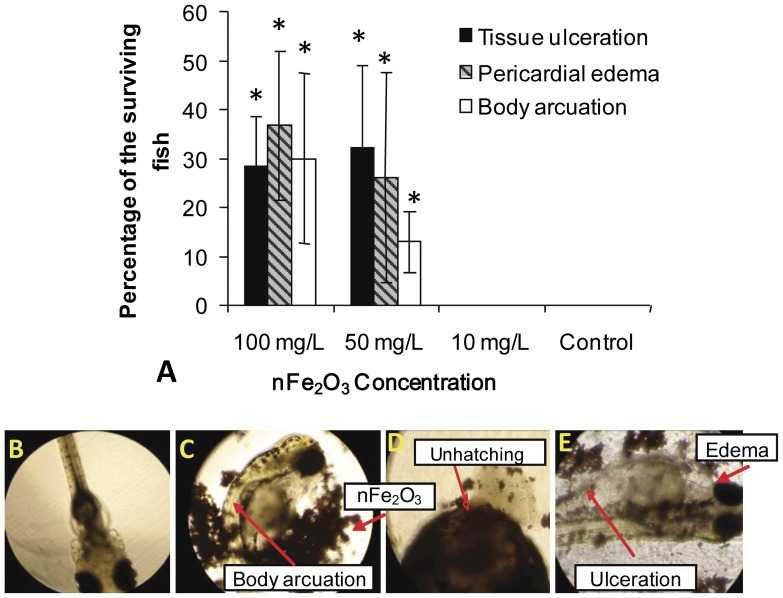
Malformations (e.g., pericardial edema, tissue ulceration, and body arcuation) induced by nFe_2_O_3_ at 168 hpf. (A) Malformation percentage in the surviving fish; (B) control fish; (C) hatching fish with body arcuation, treated with 50 mg/L of nFe_2_O_3_ aggregates; (D) unhatching embryos, treated with 50 mg/L of nFe_2_O_3_ aggregates, dead at 168 hpf; (E) hatching fish with pericardial edema, treated with 100 mg/L of nFe_2_O_3_ aggregates. Error bars represent ± one standard deviation from the mean of three replicates. Significance indicated by: *p<0.05.

In this study, malformation mediated by nFe_2_O_3_ in the embryos and larvae from both the control and treatment groups was recorded. Malformation was not found in zebrafish embryos or larvae exposed to nFe_2_O_3_ at a concentration of ≤10 mg/L during the 168-h exposure time (Figure 3A). However, at a concentration of >50 mg/L, the embryos and larvae exhibited severe malformations, characterized by tissue ulceration, pericardial edema, and body arcuation (Figure 3). Some affected embryos were unable to hatch and eventually died (Figure 3). In the 50-mg/L treatment group, 12.5%, 25%, and 32.5% of the surviving embryos and larvae showed significant (*p*<0.05) tissue ulceration, pericardial edema, and body arcuation, respectively. Malformation was more serious in the embryos and larvae exposed to 100 mg/L nFe_2_O_3_, although no significant difference was found from those in the 50-mg/L treatment group.

### Developmental toxicity of iron oxide NPs

In this study, the addition of nFe_2_O_3_ to the culture medium resulted in the formation of aggregates that settled out of the water column very quickly. This aggregation and sedimentation phenomenon of nFe_2_O_3_ is similar to that of other NPs, including Cu, Ag, TiO_2_, nZnO, nAl_2_O_3_, fullerene NPs, and single-walled carbon nanotubes (SWCNTs) [32]–[39]. These findings revealed that aggregates or agglomerates of NPs are likely to settle out of solution and sink into sediments rather than remain in suspension. Thus, benthic organisms living in sediments or at the bottom of aquatic environments could be potential targets of NPs released into the environment. Zebrafish embryos are demersal and can settle to the bottom of the water column, which allows a mimicking of the direct contact between benthic biota and NPs in the sediment. Therefore, zebrafish embryos may serve as an effective model to explore the potential ecological effects underlying the toxicity of NP aggregates that settle out of the water column. In our experiment, nFe_2_O_3_ aggregates (≥10 mg/L) were found to be toxic to zebrafish embryos and larvae, causing a dose-dependent mortality and hatching inhibition (Figure 2). Developmental abnormalities, such as pericardial edema, malformation, and tissue ulceration, were also found in the group exposed to 50 and 100 mg/L of nFe_2_O_3_ (Figure 3); these affected more than 12.5% of the surviving fish by 168 hpf. To our knowledge, the present study is one of the first to evaluate developmental toxicity in vertebrate fish caused by exposure to nFe_2_O_3_ in aquatic environments. Previous studies have shown that doses higher than 50 mg/kg of dimercaptosuccinic acid-coated iron oxide (Fe_3_O_4_) NPs can disrupt mouse embryo development, causing a significant decrease in the growth of infant animals [11]. Moreover, Nations et al. (2011) demonstrated that although exposure to nFe_2_O_3_ (0.001 mg/L to 1000 mg/L) caused no mortality or significant malformation in frog (*Xenopus laevis*) embryos and larvae, nFe_2_O_3_ exposure did decrease tadpole SVL even under a low concentration of 0.001 mg/L [23]. These studies demonstrate that organisms in the early stages of embryonic development are usually more sensitive to toxicological effects. Thus, examining organisms at these stages can help evaluate the sublethal effect of NPs and distinguish the nature of the toxicological effect (e.g., neural toxicity and genotoxicity) [40], [41].

### Toxicity assessment of NPs using the ELS test

The reported results relating to the toxicity assessment of iron oxide NPs are disputed. In general, iron oxide NPs have been widely used in several fields, and they are considered non-toxic materials [24], [42]–[44]. Kim et al. (2006) suggested that iron oxide NPs do not cause apparent toxicity to mice *in vivo* [44]. However, other researchers have reported severe toxicity in the cell system or *in vivo* model of rodents [11], [17], [18], [45]–[47]. For example, nFe_2_O_3_ was able to induce lung injury in rats, increase microvascular permeability and cell lysis in lung epithelia, and significantly disturb blood coagulation parameters [17]. Further investigation showed that nFe_2_O_3_ entered the central nervous system, induced severe oxidative stress, and damaged nerve cells in mice [18]. The toxic effects of NPs in the environment depend on initial physicochemical properties (such as composition, size, additives, specific surface area, surface charge, and synthesis method employed), environmental factors, test organisms, and experimental methods [48]–[50]. Thus, a basic set of tests is warranted to determine the toxicity of certain NPs to extract reliable conclusions about their toxicological effects. Therefore, the ELS test (used in this study) using zebrafish embryos/larvae as an animal model may serve as a good protocol to explore the potential mode of action underlying the toxicity of NP aggregates. This model offers several advantages [31], [38], [40], [51]. First, zebrafish embryos are demersal: they settle to the bottom of the water column and make direct contact with sediments, mimicking the direct contact between the biota present in the sediments and the NPs that settle out of the water column. Second, transparency and extra-uterine development can be examined, allowing direct observation of phenotypic changes during embryonic development. Third, zebrafish share many cellular and physiological characteristics with higher vertebrates. Toxicological results can thus be compared with those from studies on developmental toxicity in mammals. Fourth, the zebrafish embryo model has been employed to study the ecotoxicology of other biohazards. Therefore, although no international consensus exists about which toxicity tests should be used for NP toxicity assessment [24], the results from the ELS test may provide useful data for assessing the potential environmental effects and health risks of NPs.

### Mechanism of toxic effects of iron oxide NPs

Little information exists on the toxic effects of iron oxide NPs [8], and to our knowledge there is no published study on the developmental toxicity of nFe_2_O_3_ in aquatic organisms. The impact of nFe_2_O_3_ on zebrafish development observed in this study was found to be associated with the aggregation and sedimentation of nFe_2_O_3_ and with the characteristics of nanoparticles.

Accompany the aggregation and sedimentation of nFe_2_O_3_, direct adherence/adsorption of nFe_2_O_3_ aggregates could be observed on the surface of embryos (Fig. 3). In a previous study, we found that this direct adherence/adsorption of NPs may exert a physical effect on experimental embryos, causing toxicity [52]. For example, the direct adherence/adsorption of nFe_2_O_3_ aggregates on the surface may cause hatching delay of embryos through a change in the surface mechanical properties or by interfering with the digestive function of the chorionic hatching enzyme [33]. He et al. showed that iron oxide nanoparticles located on the surface of *Escherichia coli* damaged the cell wall and outer membrane [53]. Moreover, direct adherence/adsorption may also interfere with nutrient exchange between the embryos and the environment. For example, the direct adherence/adsorption of nFe_2_O_3_ aggregates on the surface may cause depletion of oxygen exchange, resulting in hypoxia of embryos on exposure; this has been reported to cause delayed hatching and development of embryos [33]. In addition, the adherence and/or adsorption of nFe_2_O_3_ aggregates may also cause excessive production of reactive oxygen species (ROS) (i.e., NO and O_2_^•−^) *in vivo*, resulting in oxidative stress for the embryos, which may be critical in inducing the observed developmental toxicity [38], [54].

Another crucial factor that may have affected the zebrafish embryos is the release of metal ions from the nanoparticles. The aggregation and sedimentation of nFe_2_O_3_ may lead to a high localized concentration. Given the direct adherence/adsorption of nFe_2_O_3_ aggregates on the embryo surface, there could be high levels of free iron ions in the exposed tissue. This iron overload could thus have toxic implications as an excessive accumulation of nFe_2_O_3_. In particular, it could lead to an imbalance in homeostasis and aberrant cellular responses, including cytotoxicity, DNA damage, oxidative stress, epigenetic events, and inflammatory processes, which would eventually lead to the observed toxicity [55]. He et al. confirmed the dissolution of iron oxide nanoparticles and demonstrated that the iron ion and uptake of nanoparticles facilitated iron binding with proteins and DNA strands, resulting in greater mutation frequency in *E. coli* [53]. We previously demonstrated that zinc oxide particle (nZnO) toxicity may be attributed to both the nZnO and the released Zn^2+^ [38]. More research is needed to evaluate the role of the direct physical effect and/or release of metal ions from nanoparticles in the case of nFe_2_O_3_-mediated toxicity.

### Iron oxide NPs in real aquatic environments

The zebrafish culture medium used in the present study is not the same as a real aquatic environment (such as lakes and rivers), and in a real environment nFe_2_O_3_ may behave differently. Keller et al. reported that nanoscale titanium dioxide particles (nTiO_2_), nZnO, and cerium oxide particles (nCeO_2_) were relatively stable in natural freshwater: over 90% of these nanomaterials remained in the water body even after standing for 6 h at a concentration of 200 mg/L [56]. However, in seawater, less than 30% of these nanomaterials remained after 6 h [56]. Kádár et al. examined the aggregation and sedimentation of nFe_2_O_3_ in natural seawater: ≤30% of nFe_2_O_3_ remained in the seawater after 12 h [8]. Thus, the conditions in the present study were those of an “ideal” experimental situation, using standard zebrafish culture medium as a simulated aquatic environment. This was done to determine whether traditionally standardized tests, such as the ELS test, are useful in determining the ecotoxicity of NPs when no or insufficient data are available. In addition, such ideal experimental conditions allow comparisons of studies among different research groups.

Since there have been no major releases of nFe_2_O_3_, little is known about the distribution level of the metal oxide NPs in real aquatic environments—except for nTiO_2_, which has been detected at over 0.1 mg/L (calculated as Ti) in the runoff from an urban area of Switzerland [57]–[59]. Furthermore, Klaine et al. pointed out that for some kinds of NPs, such as iron oxide NPs, a large background of naturally occurring iron in the dissolved phase exists; this makes it difficult to differentiate natural from manufactured material [58]. However, nanotoxicology is a new field, and more data are needed with respect to risk assessment and management; examining whether NPs may be toxic to aquatic organisms is an important first step. The present study, which covered a wide range of nFe_2_O_3_ concentrations from 0.1 to 100 mg/L, was intended to address this issue and hopefully provide some guidance regarding risk assessment and acceptable safe exposure levels. Though much work remains ahead, this paper is the first report on the developmental toxicity of nFe_2_O_3_ in an aquatic organism. Therefore, this study should serve as the basis for future research into chemical and physical factors that control the toxicity of such nanomaterials as well as a basis for further studies to determine the necessary data to set water-quality standards to protect aquatic life. Such research will benefit risk assessment and management efforts and may contribute indirectly to the development of nanotechnology.

## Conclusions

With the increasing use of metal oxide nanomaterials in catalysis, sensors, environmental remediation, and such commercial products as ones for personal care, there is a strong possibility that these nanomaterials will ultimately enter aquatic ecosystems through wastewater discharge and washing off during recreational activities, such as swimming and water skiing, thereby impacting on the environment and human health. Our results in this study demonstrate that nFe_2_O_3_ aggregates caused a serious delay in embryo hatching, malformation in some zebrafish embryos and larvae, and eventually mortality. The results of this work will contribute to our understanding of the potential ecotoxicological impact of NPs released to aquatic environments. Moreover, our results also suggest that the ELS test using zebrafish embryos/larvae could be a standard method for assessing the potential toxicity of NPs, especially in consideration of the fact that NPs could accumulate in sediments.
